# Prediction of DtxR regulon: Identification of binding sites and operons controlled by Diphtheria toxin repressor in *Corynebacterium diphtheriae*

**DOI:** 10.1186/1471-2180-4-38

**Published:** 2004-09-24

**Authors:** Sailu Yellaboina, Sarita Ranjan, Prachee Chakhaiyar, Seyed Ehtesham Hasnain, Akash Ranjan

**Affiliations:** 1 Computational and Functional Genomics Group, Centre for DNA Fingerprinting and Diagnostics, Hyderabad 500076, INDIA; 2 Laboratory of Cellular and Molecular Biology, Centre for DNA Fingerprinting and Diagnostics, Hyderabad 500076, INDIA

## Abstract

**Background:**

The diphtheria toxin repressor, DtxR, of *Corynebacterium diphtheriae *has been shown to be an iron-activated transcription regulator that controls not only the expression of diphtheria toxin but also of iron uptake genes. This study aims to identify putative binding sites and operons controlled by DtxR to understand the role of DtxR in patho-physiology of *Corynebacterium diphtheriae*.

**Result:**

Positional Shannon relative entropy method was used to build the DtxR-binding site recognition profile and the later was used to identify putative regulatory sites of DtxR within *C. diphtheriae *genome. In addition, DtxR-regulated operons were also identified taking into account the predicted DtxR regulatory sites and genome annotation. Few of the predicted motifs were experimentally validated by electrophoretic mobility shift assay. The analysis identifies motifs upstream to the novel iron-regulated genes that code for Formamidopyrimidine-DNA glycosylase (FpG), an enzyme involved in DNA-repair and starvation inducible DNA-binding protein (Dps) which is involved in iron storage and oxidative stress defense. In addition, we have found the DtxR motifs upstream to the genes that code for sortase which catalyzes anchoring of host-interacting proteins to the cell wall of pathogenic bacteria and the proteins of secretory system which could be involved in translocation of various iron-regulated virulence factors including diphtheria toxin.

**Conclusions:**

We have used an *in silico *approach to identify the putative binding sites and genes controlled by DtxR in *Corynebacterium diphtheriae*. Our analysis shows that DtxR could provide a molecular link between Fe^+2^-induced Fenton's reaction and protection of DNA from oxidative damage. DtxR-regulated Dps prevents lethal combination of Fe^+2 ^and H_2_O_2 _and also protects DNA by nonspecific DNA-binding. In addition DtxR could play an important role in host interaction and virulence by regulating the levels of sortase, a potential vaccine candidate and proteins of secretory system.

## Background

Iron is an important inorganic component of a cell. Iron is required as co-factor for various essential enzymes and proteins some of which are involved in electron transport (Cytochromes), redox reactions (oxidoreductases) and regulation of gene expression (fumarate-nitrate reduction regulatory protein, iron-binding protein) [1]. However a higher level of intracellular iron can catalyze formation of hydroxyl radicals and reactive oxygen species through Fenton's reaction which could be lethal to the cell [2]. Hence, a careful regulation of iron-requiring enzymes/proteins and iron uptake proteins/enzymes is required for the survival of bacteria.

Inorganic iron is also known to influence virulence in many pathogenic bacteria such as *Corynebacterium diphtheriae*, *Escherichia coli*, and *Bordetella bronchiseptica *[3-5]. The diphtheria toxin repressor DtxR is known as an iron-activated global transcription regulator that represses the transcription of various iron-dependent genes in *C. diphtheriae *[6,7]. Eight DtxR-binding sites in upstream sequences of operons/genes named as *tox, hmuO, irp1, irp2, irp3, irp4, irp5 and irp6 *have been identified by DNA footprinting methods [6]. The product of *tox *gene is diphtheria toxin which catalyzes the NAD-dependent ADP ribosylation of eukaryotic aminoacyl-transferase-II, thereby causing inhibition of protein synthesis and subsequent death of the host. The *hmuO *gene, which encodes a haem oxygenase, oxidizes the haem to release free iron. The operons *irp1 *and *irp6 *encode the products with homology to ABC-type ferric-siderophore transport systems. The gene *irp3 *encodes a homologue of AraC-type transcriptional activators. The products of *irp2, irp4 and irp5 *do not show any homology to the other known proteins. In addition, *C. diphtheriae *with inactive DtxR has been shown to be sensitive to killing by exposure to high iron conditions or hydrogen peroxide than the wild type [8].

This work uses an *in silico *method to identify additional DtxR-binding sites and target genes to understand the role of DtxR in virulence and patho-physiology of *C. diphtheriae*.

## Results

### *In silico *identification of putative DtxR-binding sites

Experimentally characterized DtxR-binding motifs were collected from the literature (Table 1). These binding sites were used to identify additional putative DtxR-binding sites along with associated operons in C. *diphtheriae *NCTC13129 genome (see materials and methods). Table 2 shows the predicted DtxR-binding sites with score 3.7438 or more. We could identify five (tox, irp4, irp5, irp6 and hmuO) of the eight known DtxR-binding sites, in sequenced *C. diphtheriae *NCTC13129 genome. We could not find irp1 and irp2 motifs as the corresponding genes (*irp1, irp2*) are not present in the sequenced strain NCTC13129 [9]. The regulator binding sites of *irp3*, *irp4 *and *irp6 *genes in the strain NCTC13129 shows one base change from the binding sites reported in strain C7 [6]. Binding site of *irp3 *gene (TTAGGTGAGACGCACCCAT) although exists in strain NCTC13129, but not there in the predicted sites, because it is located within the coding region of *irp3 *ORF. The predicted ORF of *irp3 *in the sequenced strain NCTC13129 has different start position and is larger than what was previously reported in strain C7 [9,10].

In addition, we have identified binding sites in upstream sequences of eight genes recently reported to be regulated by DtxR [7]. However, our prediction differs from the previous report for five (secY, deoR, chtA, frgA, sidA) of the seven sites which were identified by BLAST search (Table 2). Our prediction agreed with the previous report that the genes such as *recA *(DIP1450) and *ywjA *(DIP1735) are not under a direct DtxR regulation as we could not detect any motif upstream to these gene with scores above the cutoff value [7].

### Experimental validation of predicted binding sites

Since our approach to identify DtxR-regulated genes is purely computational in nature, we decided to test the validity of our predictions. A sample of predicted regulator binding motifs (Table 2) (upstream to ORFs: DIP2161, DIP0699, DIP0586, DIP2304, DIP2272) were experimentally verified by EMSA using IdeR, an orthologue of DtxR from *M. tuberculosis*. DtxR and IdeR are iron-dependent regulators. A pair wise sequence comparison of the two proteins shows a high (58%) overall sequence identity (similarity 72%) which increases further to 92% identity and 100% similarity in DNA recognition domain. In addition, the structural comparison of two regulators also shows a very similar 3D organization, suggesting that the IdeR regulator would be able to recognize the DtxR motif [11].

**Figure 1:**
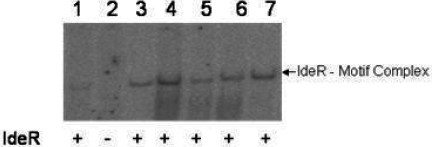
IdeR binds the predicted DtxR-binding DNA fragments. 30 pmoles of IdeR was added to ^32^P-labelled DNA probes in the presence of 200 μM Mn^2+^, and complexes were resolved on a 7% Tris-borate polyacrylamide gel containing 150 μM Mn^2+^. Lane 1: Control gel retardation using Radiolabeled DNA motif without DtxR-binding site. Lane 2: Radiolabeled DIP2161 motif without IdeR. Lane 3: Radiolabeled DIP2161 motif with IdeR. Lane 4: Radiolabeled DIP0699 motif with IdeR. Lane 5: Radiolabeled DIP0586 motif with IdeR. Lane 6: Radiolabeled DIP2304 motif with IdeR. Lane 7: Radiolabeled DIP2272 motif with IdeR.

Synthetic double stranded oligonucleotides corresponding to DNA-binding sites were labeled with ^32^P and mixed with purified IdeR in presence of manganese ions and was assayed for the formation of DNA-protein complex using EMSA. Manganese was used as the divalent metal in the binding reactions on account of its redox stability compared with ferrous ion. Electrophoretic mobility of all five double stranded oligonucleotides tested was retarded by IdeR (Figure 1). However a synthetic motif (TTTTCATGACGTCTTCTAA) used as a negative control did not show any complex formation. These results indicate that the predicted DtxR-binding sites can indeed bind to DtxR.

### Identification and annotation of DtxR-regulated genes *C. diphtheriae genome*

In addition to the binding site prediction, we have also identified co-regulated genes (operons) downstream to the predicted DtxR-binding site (Table 3). Function of the proteins encoded by the putative genes in Table 2 and Table 3 was predicted by RPS-BLAST search against conserved domain database [12].

## Discussion

Our analysis identified putative DtxR motifs upstream to various operons/genes which could be involved in siderophore biosynthesis, ABC-type transport systems, iron storage, oxidative stress defense and iron-sulfur cluster biosynthesis. In addition, we have also identified the motifs upstream of operons that could be involved in anchoring of host-interacting proteins to the cell wall and secretion of various virulence factors. Important functions of some of these DtxR-regulated genes and their role in *C. diphtheriae *physiology are discussed here.

### Regulation of siderophore biosynthesis and ABC-type transport systems

Predicted member of the DtxR regulon, the gene DIP0586, codes for the IucA/IucC family of enzymes that catalyze discrete step in the biosynthesis of the aerobactin [13]. In addition to known DtxR-regulated siderophore transport genes (irp1, irp6), DtxR could also regulate other ABC-type transport systems similar to Manganese/Zinc, peptide/Nickel and multidrug subfamilies of ABC transporters. The peptide/nickel transport system (DIP2162-DIP2165) has been suggested to be recently acquired by pathogenic *C. diphtheriae *[9].

### Regulation of iron storage and oxidative stress defense

We predict that DtxR could regulate divergently transcribed genes DIP2303 and DIP2304 whose products are similar to starvation inducible DNA-binding protein (Dps) and Formamidopyrimidine-DNA glycosylase (Fpg), respectively. Dps in *Escherichia coli *is induced in response to oxidative or nutritional stress and protects DNA from oxidative stress damage by nonspecific binding [14]. Dps also catalyzes oxidation of ferrous iron to ferric iron by hydrogen peroxide (2Fe^2+ ^+ H_2_O_2 _+ 2H_2_O → 2Fe^+3^OOH_(core) _+ 4H^+^) which in turn prevents hydroxyl radical formation by Fenton's reaction (Fe^2+ ^+ H_2_O_2 _→ Fe^+3 ^+ HO^- ^+ HO^.^) and thereby prevents subsequent DNA damage [15]. The enzyme, formamidopyrimidine-DNA glycosylase is a primary participant in the repair of 8-oxoguanine, an abundant oxidative DNA lesion [16]. The gene DIP1510 which codes for the site-specific recombinase XerD could also be regulated by DtxR. The *xerD *gene in *E. coli *belongs to the oxidative stress regulon [17].

### Regulation of proteins involved in iron-sulfur cluster biosynthesis and iron-sulfur cluster containing proteins

We predict that the operon DIP1288-DIP1296, which is similar to the *suf *operon of *E. coli*, could be regulated by DtxR. The *suf *operon in bacteria encodes the genes for Fe-S cluster assembly machinery [18]. In addition, genes encoding the iron-sulfur containing proteins such as succinate dehydrogenase (Sdh), cytochrome oxidase (CtaD) and Ribonucleotide reductase (NrdF1) in *C. diphtheriae *also show DtxR motif in their upstream sequences.

### Regulation of sortases

We predict that DtxR could regulate the recently acquired pathogenic island DIP2271-DIP2272, encoding the sortase srtA and hypothetical protein, respectively [9]. Sortases are membrane-bound trans-peptidases that catalyze the anchoring of surface proteins to the cell wall peptidoglycan [9]. Such systems are often used by gram-positive pathogens to anchor host-interacting proteins to the bacterial surface [19].

### Regulation of protein translation and translocation system

DtxR could regulate two operons that contain genes DIP0699 (*secA*) and DIP0540 (*secY*) that code for the protein translocation system. The *sec*Y-containing operon, which is similar to the streptomycine operon spc from *B. subtilis *and other bacteria, involves the genes required for protein translation and translocation [20]. The operon contains additional sialidase gene (DIP0543) in comparison to non pathogenic Corynebacterium species. Activity of sialidase has been linked to virulence in several other microbial pathogens and may enhance fimbriae mediated adhesion in *Corynebacterium diphtheriae *by unmasking receptors on mammalian cells [9].

The Sec system can both translocate proteins across the cytoplasmic membrane and insert integral membrane proteins into it. The former proteins but not the latter possess N-terminal, cleavable, targeting signal sequences that are required to direct the proteins to the Sec system. Some of the DtxR-regulated genes including diphtheria toxin (Table 4) show predicted signal sequences by SignalP 3.0 [21] and hence they may play an important role in host interaction and virulence of *Corynebacterium diphtheriae *[9].

## Conclusions

The bioinformatics method used to predict the targets of DtxR in *C. diphtheriae *NCTC13129 genome is promising, as some of the predicted targets were experimentally verified. The approach identified novel DtxR-regulated genes, which could play an important role in physiology of *C. diphtheriae *NCTC13129. DtxR, generally known as a repressor of diphtheriae toxin and iron siderophore/transport genes, can also regulate other metal ion transport genes, iron storage, oxidative stress, DNA-repair, biosynthesis of iron-sulfur cluster, Fe-S-cluster containing proteins, and even protein sortase and translocation systems.

## Methods

### Source of genome sequence

The complete genome sequence of *C. diphtheriae *was downloaded from NCBI ftp site [22], and the DtxR-binding sites identified by experimental methods were collected from literature [6,10,25-27].

### Prediction of DtxR-binding sites

DtxR-binding site recognition profile was calculated by positional Shannon relative entropy method [23,24]. The positional relative entropy *Q*_*i *_at position *i *in a binding site is defined as


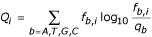


where *b *refers to each of the possible base (A, T, G, C), *f*_*b,i *_is observed frequency of each base at position *i *and *q*_*b *_is the frequency of base *b *in the genome sequence. The contribution of each base to the positional Shannon's relative entropy is calculated by multiplying positional frequency of each base with positional relative entropy. The binding site profile thus generated was used to scan upstream sequences of all the genes of the *Corynebacterium diphtheriae *genome. The score of each site is calculated as the sum of the respective positional Shannon relative entropy of each of the four possible bases. A maximally scoring site is selected from the upstream sequence of each gene. The lowest score among the input binding sites is considered as cut-off score. The sites scoring higher than the cut-off value are reported as potential binding sites conforming to the consensus sequence.

### Prediction of operons

Co-directionally transcribed genes, downstream to the predicted binding site were selected as potential co-regulated genes (operons) according to one of the following criteria (a) Co-directionally transcribed orthologous gene pairs, conserved in at least 4 genomes; (b) genes belong to the same cluster of orthologous gene function category and the intergenic distance is less than 200 base pairs; (c) the first three letters in gene names are identical (gene names for putative genes were assigned from COG database); (d) intergenic distance is less than 90 base pairs [24].

### Functional assignment of genes

The function of predicted genes was inferred using the RPS-BLAST search against conserved domain database [12]. These genes were further classified according to their function.

### Expression and purification of IdeR

The iron-dependent regulator IdeR from M. *tuberculosis *was expressed from a recombinant pRSET vector containing the IdeR gene fused to a six His affinity tag (P. Chakhiyar unpublished). The expressed protein was first purified using Ni-NTA Metal Chelate Affinity chromatography; later it was desalted and concentrated using Centricon Ultra filtration device. The concentration of the recombinant protein was estimated using Bradford method.

### Electrophoretic mobility shift assay

Double-stranded oligonucleotides containing the predicted binding motif (19 bp long) were end labeled with T4 polynucleotide kinase and [γ^32^P]-ATP and were incubated with the recombinant purified IdeR protein in a binding reaction mixture. The binding reaction mixture (20-μl total volume) contain the DNA-binding buffer (20 mM Tris-HCl [pH 8.0], 2 mM DTT, 50 mM NaCl, 5 mM MgCl_2_, 50% glycerol, 5 μg of bovine serum albumin per ml), 10 μg of poly(dI-dC) per ml (for nonspecific binding) and 200 μM MnCl_2_. The reaction mixture was incubated at room temperature for 30 min. Approximately 2 μl of the tracking dye (50% sucrose, 0.6% bromophenol blue) was added to the reaction mixture at the end of incubation and was loaded onto 7% polyacrylamide gel containing 150 μM MnCl_2 _in 1 × Tris-borate-EDTA buffer. The gel was electrophoresed at 200 V for 2 hours. Subsequently the gel was dried and exposed to Fuji Storage Phosphor Image Plates for 16 hours. The image plates were subsequently scanned in Fuji Storage Phosphor Imaging workstation.

## List of abbreviations

DtxR – Diphtheria toxin repressor; IdeR – Iron-dependent regulator; Dps – DNA-binding protein from starved cells; RPS-BLAST – Reversed Position Specific – Basic Local Alignment Search Tool; EMSA – Electrophoretic Mobility Shift Assay

## Authors' contributions

SY: carried out the computation, data analysis, and manuscript preparation. SR: Carried out the EMSA and drafted the manuscript. PC: provided the cloned IdeR construct, drafted the manuscript. SH: Manuscript preparation and coordination. AR: Design of the study and coordination. All authors read and approved the final manuscript.

## Figures and Tables

**Table 1:** Known DtxR-binding sites from *C. diptheriae*

**Binding site**	**Gene**	**Product**	**Reference**
TTAGGATAGCTTTACCTAA	*tox*	Diphtheria toxin	[25]
TTAGGTTAGCCAAACCTTT	*Irp1*	Periplasmic protein of siderophore transport system	[26]
GCAGGGTAGCCTAACCTAA	*Irp2*	Hypothetical protein	[26]
TTAGGTGAGACGCACCCAT	*Irp3*	AraC-type transcription regulator	[10]
ATTACTAACGCTAACCTAA	*Irp4*	Hypothetical protein	[10]
CTAGGATTGCCTACACTTA	*Irp5*	Hypothetical protein	[10]
TTTCCTTTGCCTAGCCTAA	*Irp6*	Periplasmic protein of siderophore transport system	[6]
TGAGGGGAACCTAACCTAA	*hmuO*	Haem oxygenase	[27]

**Table 2:** Predicted DtxR-binding sites in *C. diphtheriae*

**Score**	**Position**	**Site**	**Gene**	**Synonym**	**Product**
4.45904	-80	TGAGGGGAACCTAACCTAA	*hmuO*	DIP1669**	heme oxygenase
4.39003	-52	TTAGGATAGCTTTACCTAA	*Tox*	DIP0222**	Diphtheria toxin precursor
4.25877	-60	ATAGGCTACACTTACCTAA	-	DIP0624	Putative membrane protein
4.21068	-168	TTGGATTAGCCTACCCTAA	-	DIP2162**	ABC-type peptide transport system periplasmic component
4.2033	-21	*TTAGGGTAGCTTCGCCTAA*	*iucA*	DIP0586	Putative siderophore biosynthesis related protein
4.17632	-78	ATAGGCATGCCTAACCTCA	-	DIP2330	Putative membrane protein
4.07921	-130	TTAGGTCAGGGTACCCTAA	-	DIP0370	Putative succinate dehydrogenease cytochrome B subunit
4.03559	-30	TTAGCTTAACCTTGCCTAT	*arsR*	DIP0415	Putative ArsR family regulatory protein
4.01967	-239	*TTAGGGTAGGCTAATCCAA*	sidA*	DIP2161	nonribosomal peptide synthase
3.99985	-74	TTTTCTTTGCCTAGCCTAA	*irp6A*	DIP0108**	Ferrisiderophore receptor Irp6A
3.99195	-241	TTAGGCACCCCTAACCTAG	-	DIP0539	Putative sugar ABC transport syste ATP-binding protein
3.98554	-72	TTAGCTTAGCCCTAGCTAA	-	DIP0169	Putative secreted protein
3.9296	-26	CTAGGATTGCCTACACTTA	*Irp5*	DIP0894**	Conserved hypothetical protein
3.9073	-93	GTTGGGTTGCCCAACCTAC	-	DIP2106	Putative ABC transport system, ATP-binding subunit
3.89763	-86	ATAGGTTAGGTTAACCTTG	chtA*	DIP1520	Putative membrane protein
3.89676	-130	*TTGTGTTAGCCTAGGCTAA*	*secA*	DIP0699	Translocase protein
3.89169	-26	*TTGGGGTGGCCTATCCTTA*	-	DIP2304	Putative DNA-repair glycosylase
3.88042	-172	TTAGGTAAGTGTAGCCTAT	htaA*	DIP0625	Putative membrane protein
3.86534	-69	ATTACTAATGCTAACCTAA	*Irp4*	DIP2356**	Putative conserved membrane protein
3.85539	-173	TTAGGGTGGGCTAACCTGC	deoR*	DIP1296	Putative DNA-binding protein
3.84889	-75	TTAGGGAACTCTTGCCTTA	piuB*	DIP0124	Putative membrane protein
3.83816	-121	TTAGCTAGGGCTAAGCTAA	-	DIP0168	Putative glycosyl transferase
3.83576	-219	GTAACAAAGGCAAGCCTAA	*xerD*	DIP1510	Putative integrase/recombinase
3.8224	-216	ATAGGCAAGGTTAAGCTAA	-	DIP0417	Putative membrane protein
3.81905	-47	GTTGGACAGGTTACCCTAA	frgA*	DIP1061	Putative iron-siderophore uptake system permease
3.8148	-37	*TGTGGGCACACCAACCTAA*	-	DIP2272	possible sortase-like protein
3.76235	-136	TTGGGGTTGCCCTTCCTAA	-	DIP0142	Hypothetical protein
3.76233	-268	CTAGGTTAGGGGTGCCTAA	secY*	DIP0540	preprotein translocase SecY subunit
3.74673	-110	TAAACATAGCCAAACCAAA	*nrdF1*	DIP1865	ribonucleotide reductase beta-chain 1
3.7438	-81	TAAGGATAGGCCACCCCAA	*Dps*	DIP2303	Starvation inducible DNA-binding protein

Note: **Indicate the gene synonym with experimentally identified binding site in *C. diphtheriae *[6]. * Indicates the genes known to be regulated by DtxR [7]. The binding sites in Italics were verified by EMSA. The gene pairs, DIP0624-DIP0625, DIP2161-DIP2162, DIP0168-DIP0169, DIP0539-DIP0540 and DIP2303-DIP2304 are divergently transcribed and contain common regulatory regions.

**Table 3:** Predicted DtxR-regulated operons in *C. diphtheriae*

**Synonym**	**Gene**	**Orthologue**	**Product**
DIP2158		COG1131	ABC-type transport system permease and ATPase component
DIP2159		COG1131	ABC-type transport system permease and ATPase component
DIP2160	-	COG3321	Polyketide synthase modules and related proteins
DIP2161*	-	COG1020	Non-ribosomal peptide synthetase modules and related proteins

DIP0586	*iucA*	Pfam04183	Catalyse discrete steps in biosynthesis of the siderophore aerobactin
DIP0587	-	-	Putative membrane protein
DIP0588	-	-	Putative membrane protein

DIP1059	*fepC*	COG1120	ABC-type cobalamin/Fe3+-siderophores transport systems
DIP1060	*fepG*	COG4779	ABC-type enterobactin transport system
DIP1061*	*fepD*	COG0609	ABC-type Fe3+-siderophore transport system

DIP2162	*ddpA*	COG0747	ABC-type peptide transport system periplasmic component
DIP2163	*ddpB*	COG0601	ABC-type peptide/nickel transport systems permease components
DIP2164	*ddpC*	COG1173	ABC-type peptide/nickel transport systems permease components
DIP2165	*dpdD*	COG0444	ABC-type peptide/nickel transport systems ATPase component

DIP0169	*lraI*	COG0803	ABC-type metal ion transport system, periplasmic component
DIP0170	*znuC*	COG1121	ABC-type Mn/Zn transport systems, ATPase component
DIP0171	*znuB*	COG1108	ABC-type Mn2+/Zn2+ transport systems, permease components
DIP0172	*znuB*	COG1108	ABC-type Mn2+/Zn2+ transport systems, permease components
DIP0173	*lraI*	COG0803	ABC-type metal ion transport system, periplasmic component

DIP2106	*mdlB*	COG1131	ABC-type multidrug transport system, ATPase and permease component
DIP2107	*mdlB*	COG1131	ABC-type multidrug transport system, ATPase and permease component

DIP0625	*htaa*	Pfam04213	Haemin transporter associated protein
DIP0626	*hmuT*	COG4558	ABC-type haemin transport system
DIP0627	*hmuU*	COG0609	ABC-type Fe3+-siderophore transport system
DIP0628	*hmuV*	COG4559	ABC-type haemin transport system
DIP0629*	*htaa*	Pfam04213	Haemin transporter associated protein

DIP1519*	*htaa*	pfam04213	Haemin transporter associated protein
DIP1520*	*htaa*	pfam04213	Haemin transporter associated protein

DIP2303	*dps*	COG0783	Starvation inducible DNA-binding protein

DIP2304	-	COG0266	Formamidopyrimidine-DNA glycosylase
DIP2305	-	COG0063	Predicted sugar kinase

DIP1510	*xerD*	COG4974	Site-specific recombinase

DIP1288	-	-	Conserved hypothetical protein
DIP1289	*uup*	COG0488	ATPase components of ABC transporters with duplicated ATPase domains

DIP1290	-	COG2151	Predicted metal-sulfur cluster biosynthetic enzyme
DIP1291	*iscU*	COG0822	NifU homolog involved in Fe-S cluster formation
DIP1292	*csd*	COG0520	Selenocysteine lyase
DIP1293	*sufC*	COG0396	ABC-type transport system involved in Fe-S cluster assembly
DIP1294	-	COG0719	ABC-type transport system involved in Fe-S cluster assembly
DIP1295	*sufB*	COG0719	ABC-type transport system involved in Fe-S cluster assembly
DIP1296*	*deoR*	COG2345	DeoR family transcriptional regulator

DIP0370	-	-	Putative succinate dehydrogenease (cytochrome b)
DIP0371	-	COG1053	Succinate dehydrogenase/fumarate reductase
DIP0372	-	COG0479	Succinate dehydrogenase/fumarate reductase
DIP0373	-	-	Putative membrane protein
DIP0374	-	-	Putative membrane protein
DIP0375	-	-	Putative membrane protein
DIP0376	-	-	Putative membrane protein
DIP0377	-	-	Putative membrane protein

DIP1864	*ctaD*	COG0843	Heme/copper-type cytochrome/quinol oxidases
DIP1865	*nrdF1*	COG0208	Ribonucleotide reductase
DIP2330	-	-	Putative membrane protein
DIP2331	-	COG1012	NAD-dependent aldehyde dehydrogenases

DIP0124*	-	Pfam03929	Uncharacterized iron-regulated membrane protein (DUF337)

DIP0622	-	-	Putative membrane protein
DIP0623	*metA*	COG2021	Homoserine acetyltransferase
DIP0624	-	-	Putative membrane protein

DIP0415	-	Pfam01022	Bacterial regulatory protein

DIP0539	-	COG3839	ABC-type sugar transport systems

DIP0168	-	-	Putative glycosyl transferase

DIP0417	-	-	Putative membrane protein

DIP0142	-	-	Hypothetical protein
DIP0143	-	-	-
DIP0144	*tra8*	COG2826	Transposase and inactivated derivatives

DIP2271	-	-	Putative membrane protein
DIP2272	-	COG3764	Sortase (surface protein transpeptidase)

DIP0699	*secA*	COG0653	Preprotein translocase subunit SecA (ATPase
DIP0700	-	-	Hypothetical protein

DIP0540*	*secY*	Pfam00344	Eubacterial secY protein
DIP0541	*Adk*	COG0563	Adenylate kinase and related kinases
DIP0542	*mapA*		Methionine aminopeptidase
DIP0543	-	-	Sialidases or neuraminidases;
DIP0544	*erfK*	Pfam03734	This family of proteins contains a conserved histidine and cysteine
DIP0545	*infA*	COG0361	Translation initiation factor 1 (IF-1)
DIP0546	*rpsM*	COG0099	Ribosomal protein S13
DIP0547	*rpsK*	COG0100	Ribosomal protein S11
DIP0548	*rpsD*	COG0522	Ribosomal protein S4 and related proteins
DIP0549	*rpoA*	COG0202	DNA-directed RNA polymerase
DIP0550	*rplQ*	COG0203	Ribosomal protein L17
DIP0551	*truA*	COG0101	Pseudouridylate synthase

Note: * Indicate the genes reported be regulated by DtxR. Genes listed together belongs to same operon.

**Table 4:** DtxR-regulated genes containing the potential signal sequence

Gene	Product
DIP0222	Diphtheria toxin
DIP0109	IRP6B
DIP2356	IRP4
DIP2162	ABC-type peptide transport system periplasmic component
DIP0172	Putative membrane protein
DIP2107	Putative integral membrane transport protein
DIP0625	Haemin transporter associated protein
DIP0626	ABC-type haemin transport system
DIP0627	ABC-type haemin transport system
DIP1519	Haemin transporter associated protein
DIP0629	Haemin transporter associated protein
DIP1520	Haemin transporter associated protein
DIP2330	Putative membrane protein
DIP0543	Sialidases or neuraminidases
